# The Endophytic Symbiont—*Pseudomonas aeruginosa* Stimulates the Antioxidant Activity and Growth of *Achyranthes aspera* L.

**DOI:** 10.3389/fmicb.2017.01897

**Published:** 2017-09-27

**Authors:** Khaidem A. Devi, Garima Pandey, A. K. S. Rawat, Gauri D. Sharma, Piyush Pandey

**Affiliations:** ^1^Department of Microbiology, Assam University, Silchar, India; ^2^Pharmacognosy and Ethnopharmacology Division, Council of Scientific & Industrial Research-National Botanical Research Institute, Lucknow, India; ^3^Bilaspur University, Bilaspur, India

**Keywords:** *Achyranthes aspera*, antioxidant, endophyte, PGPB traits, *Pseudomonas aeruginosa*

## Abstract

A plant growth promoting bacterial endophyte designated as AL2-14B isolated from the leaves of *Achyranthes aspera* L. was identified as *Pseudomonas aeruginosa* based on its phenotypic and physiological features, and 16S rRNA gene sequence analysis. AL2-14B had plant growth stimulating attributes including siderophore and indole acetic acid release, inorganic phosphate solubilization, along with nitrogenase, ammonification, and protease activities. It also exhibited antifungal property against *Rhizoctonia solani*. The plantlets grown in germ-free condition were inoculated with AL2-14B and studied for the colonization of endophyte. Significant increase in population of AL2-14B between 3rd and 5th days after inoculation was recorded. The treatment of plants with endophytic *P. aeruginosa* AL2-14B increased nitrogen, phosphorus, potassium (NPK) contents in plant by 3.8, 12.59, and 19.15%, respectively. Significant enhancement of shoot and root length, dry leaf, dry shoot and dry root weight, and leaf surface area as compared to control (*P* < 0.05) was recorded in AL2-14B inoculated plants. The antioxidant activities increased in plants grown in germ-free conditions and inoculated with AL2-14B. The present study emphasizes on the role of diazotrophic endophyte *P. aeruginosa* AL2-14B in stimulating growth of *A. aspera* L. and improvement of its medicinal properties. Significant increase in growth and antioxidant content of *P. aeruginosa* AL2-14B treated plants suggests the possibility of an economical and eco-friendly mean of achieving antioxidants rich, healthier *A. aspera* plants.

## Introduction

Endophytic bacteria colonize the interior tissue of the plant. They are ubiquitous with a rich biodiversity and unexplored biosynthetic potential (Strobel and Daisy, 2003; Ryan et al., 2009). Endophytes produce a group of bioactive compounds and enzymes to survive in the unique chemical environment of the host plant (Strobel, 2003). Their metabolic activities also help in increasing the growth and development of plants. This is because of direct growth promotion effects of endophytes, through production of plant growth regulators, N-fixation, synthesis of 1-aminocyclopropane-1-carboxylic acid (ACC) deaminase, phosphate solubilization, and/or by indirect mechanism of providing resistance to diseases through the production of antimicrobial metabolites or siderophores that inhibit pathogenic microorganisms (Sun et al., 2009; Ji et al., 2014; Abbamondi et al., 2016; Khan et al., 2016). Medicinal plants have beneficial endophyte–plant relationship that may be explored and utilized. Endophytes are considered to be a promising source of novel secondary metabolites (Schulz et al., 2002; Strobel, 2003; Puri et al., 2006) with potential for medicinal use, as well as important in agriculture and industry (Strobel and Daisy, 2003). The anticancer compound (podophyllotoxin; Puri et al., 2006) and the natural insecticide (azadirachtin; Kusari et al., 2012) produced by endophytic microorganisms are good examples for same.

The plant growth promoting properties of endophytes are unique, and therefore it is significant to study such properties from microbial populations linked with medicinally and economically important plants. *Achyranthes aspera* L (Latjeera; Rough Chaff tree) is a medicinal plant that is used for the dilation of the blood vessels, lowering of the blood pressure, depression of the heart, and increases the rate and amplitude of respiration (Neogi et al., 1970). This plant have been found effective in treating disorders like piles, renal dropsy, pneumonia, cough, kidney stone, skin eruption, snake bites, and dysentery (Aziz et al., 2005). The plant is also reported to have antiperiodic, antiasthmatic (Charyulu, 1982), diuretic (Subramaniam, 1961), purgative, laxative, hepatoprotective (Katewa and Arora, 2001), anti-allergic, and various other important medicinal properties. The pharmacological effects of *A. aspera* L. are attributed to the presence of active compound like alkaloids, saponins, sterols. Other active constituents include D-glucuronic acid, β-D-galactopyranosyl ester of D-glucuronic acid, oleanolic acid, amino acids, hentriacontane, sapogenin (Khastgir et al., 1958), ecdysterone (Ikan et al., 1971), betaine (Kapoor and Singh, 1966), *p*-benzoquinone, hydroquinone, spathulenol, nerol, α-ionone, asarone, and eugenol (Rameshwar, 2007). Considering the medicinal importance of *A. aspera*, it’s pertinent to understand the role of endophytic bacteria on its growth and other properties. Earlier, a root endophyte had been reported (Misra et al., 2012) from *A. aspera*, with focus on its phosphate solubilization activity only. In the present study, endophytic bacteria isolated from the aerial part of *A. aspera* were investigated for their plant growth promoting potential and their effect on antioxidant activity of *A. aspera*.

## Materials and Methods

### Isolation of Endophytic Bacteria

Fresh leaves of *A. aspera* L. plants were collected from different parts of Manipur, India. The leaves were washed with tap water and surface sterilized with 70% ethanol for 1 min followed by 0.1% mercuric chloride for 5 min. Leaves were washed in water, and rinsed in phosphate buffer, macerated in mortar and pestle under aseptic condition. Suitable dilution of 1.0 g of macerated tissue was plated on yeast extract mannitol agar (YEMA) and incubated at 30°C for 3 days. The bacterial colonies were selected, sub-cultured, purified, and used for further studies.

### Morphological and Biochemical Characterization of the Isolates

The isolates were characterized for morphological and biochemical properties according to Bergey’s Manual of Systematic Bacteriology (Bergey et al., 1994). Colony morphology, shape, color, and growth pattern were recorded after 24 h of growth on YEMA. The isolates were also tested for catalase (Aneja, 2006), oxidase (Cappuccino and Sherman, 1996), citrate, indole production (Seeley and VanDemark, 1981), and carbohydrate fermentation test (Aneja, 2006).

### Molecular Characterization of the Isolates

The genomic DNA of isolates was extracted using HiPurA Bacterial Genomic Purification Kit (Hi-Media). The primers 27F (5′-CAGAGTTTGATCCTGGCT-3′) and 1492R (5′-AGGAGGTGATCCAGCCGCA-3′) were used for amplification of 16S rRNA gene (Weisburg et al., 1991). The total PCR mixture was 25.0 μL comprising 2× Master mix (GCC Biotech), 12.5 μL; 27F, 1.0 μL; 1492R, 1.0 μL; lysate DNA, 1.0 μL, and nuclease free Milli-Q water, 9.5 μL. The PCR condition was an initial denaturation at 94°C for 5 min, followed by 30 cycles of 94°C for 30 s, 55°C for 30 s, and 72°C for 1.30 min and final extension at 72°C for 10 min. Five microliters of purified PCR product was loaded in the agarose gel (1%) while 1 μL DNA ladder (100 bp) (Promega) was also loaded to estimate the size of the amplified fragments. The gel was run at 100 V for about 90 min and observed under the UV transilluminator for possible amplification. The amplified 16S rRNA gene was sequenced (Xcelris Labs Ltd, India), and identified using the EzTaxon-e server database (Kim et al., 2012) and NCBI GenBank databases. It was aligned with the 16S rRNA gene sequences of other related species using CLUSTAL X v2.1 (Larkin et al., 2007). Phylogenetic analyses were performed using the software package MEGA 4 (Tamura et al., 2011).

### Pathogenicity of Isolate

The isolate *Pseudomonas aeruginosa* AL2-14B was subjected for “hemolytic activity” assay, and screening for virulence genes to check if it may be potentially pathogenic. The isolate was spot-inoculated on blood agar plates and incubated at 37°C for 48 h to determine its ability to release hemolytic cytotoxin. The plates were checked for appearance of zones for complete or partial hemolytic activity. Further, virulence genes—*toxA* (270 bp) for exotoxin and *plcH* (608 bp) for hemolysin—were screened by PCR amplification (Sabharwal et al., 2014), where 25 μL reaction mixtures had 2.5 μL of dNTPs (10 mM), 1.25 μL each of forward and reverse primers (5 pmol each), 2.5 μL 10× PCR buffer and 0.25 μL *Taq* DNA polymerase (3 μL). The following gene-specific primers were used: (a) *toxA* F (CTGCGCGGGTCTATGTGCC) and *toxA* R (GATGCTGGACGGGTCGAC), (b) *plcH* F (GCACGTGGTCATCCTGATGC) and *plcH* R (TCCGTAGGCGTCGACGTAC). PCR conditions remained as initial denaturation at 95°C for 7 min, 30 cycles of denaturation at 95°C for 1 min, annealing at 52 or 53°C for 1 min (for *toxA* or *plcH*, respectively), and extension at 72°C for 30 s, followed by a final extension at 72°C for 10 min. Clinical *P. aeruginosa* isolates—C82, A135, and C69—were used as positive control.

### Siderophore Production Assay

The ability of isolates to produce siderophore was determined by formation of orange halo around bacterial colonies on Chrome Azurol S (CAS) agar plates incubated at 30°C for 48 h (Schwyn and Neilands, 1987). The cultures were inoculated in iron-deficient CAS liquid medium (Schwyn and Neilands, 1987) and incubated on rotary shaker at 120 rpm. Quantitative estimation of siderophores was done by CAS-shuttle assay. One milliliter of culture supernatant was mixed with 1 mL of CAS reagent (10 mM HDTMA; 1 mM FeCl_3_ solution; 2 mM CAS solution) and absorbance was measured at 630 nm against a reference, having 1 mL of uninoculated broth and 1 mL of CAS reagent (Payne, 1994). The activity was recorded in “percentage siderophore units” calculated as [(Ar - As) × Ar^-1^) × 100]. Where, Ar is the absorbance of reference at 630 nm (un-inoculated media + CAS reagent) and As is the absorbance of sample at 630 nm (culture supernatant + CAS reagent). The type of siderophore produced (catecholate or hydroxamate) was determined according to Arnow’s (1937) and FeCl_3_ tests (Meyer et al., 1995).

For the determination of the threshold level of iron for siderophore production, iron content of succinic medium (Sayyed et al., 2005) was varied by the addition of ferric chloride in the range of 0–30 μM concentration. Bacterial strain was inoculated and incubated at 29°C at 120 rpm, and siderophore content was estimated as described above. The siderophore production was quantified twice, each with three replicates.

### IAA Production Assay

The indole acetic acid (IAA) production was determined by the method of Loper and Scroth (1986). The isolates were grown on YEMA medium for 5 days. A loopful of the culture was inoculated in different flasks having YEM broth, each supplemented with a different L-tryptophan concentration (0, 0.2, 0.4, 0.6, 0.8, or 1.0%) and incubated at rotatory shaker (150 rpm, 30°C). Production of IAA was measured after every 24 h interval. The cultures were harvested by centrifugation (11,000 × *g*, 15 min), 1 mL of the supernatant was mixed with 2 mL of Salkowski reagent (50 mL, 35% perchloric acid with 1 mL, 0.5 M FeCl3) (Gordon and Weber, 1951). Optical density (OD) was measured at 530 nm and the amount of IAA produced was quantified by comparing with the standard curve prepared with known concentrations of IAA. The IAA release was quantified twice, each with three replicates.

### Phosphate Solubilization, Nitrogen Fixation, and ACC Deaminase Production

The ability of isolates to solubilize inorganic phosphate was assayed using modified Pikovskaya medium (Nautiyal, 1999). The halo and colony diameter were measured every 24 h up to 4 days of incubation at 30°C. The solubilization index has been defined as the ratio of the total diameter (colony + halo zone) to the colony diameter (Premono et al., 1996). Quantitative estimation of P content in the supernatant was estimated using the vanado-molybdate colorimetric method (Koenig and Johnson, 1942).

Ability to fix atmospheric nitrogen was screened in nitrogen-free combined carbon (NFCC) supplemented with 0.5 mM glucose (Mirza and Rodrigues, 2012), where semisolid NFCC medium (0.5% agar) was inoculated with test strain and incubated under atmospheric conditions. Nitrogenase activity was determined by acetylene reduction assay to confirm nitrogen fixation ability of isolate by using GC-FID (Hardy et al., 1971). Further, *nifH* gene was amplified, with a set primer *nifH* F: 5′-CGTTTTACGGCAAGGGCGGTATCGGCA-3′ and *nifH* R: 5′-TCCTCCAGCTCCTCCATGGTGATCGG-3′. PCR conditions for the amplification of *nifH* gene fragment was denaturation at 94°C for 5 min followed by 30 cycles of denaturation at 94°C for 1 min, primer annealing at 51–57°C for 30 s, and elongation at 72°C for 1 min followed by a final step of extension at 72°C for 5 min. Amplified PCR products were resolved on 1% agarose gel. *Klebsiella pneumoniae* S4C9 was experimented in parallel for *nifH* amplification as positive control. ACC deaminase activity was screened according to El-Tarabily (2008) using the nitrogen-free Dworkin and Foster’s minimal salts agar medium (Dworkin and Foster, 1958). The solid medium was supplemented with either 2 g (NH_4_)_2_SO_4_ or 3 mM ACC per liter as sole nitrogen source, and incubated aerobically. The growth experiment was conducted twice, on five plates, at each attempt.

### Antifungal Activity

The antifungal activity of the isolates was tested *in vitro* against three strains of pathogenic fungi, i.e., *Rhizoctonia solani*, *Fusarium oxysporum*, and *Pyricularia oryzae*. The antifungal bioassays were performed using dual culture method (Khamna et al., 2009). Three days old culture was spot inoculated at the corners of the PDA plates leaving some distance from the margins. Fungal plug (6 mm) was then placed at the center of the plates. The plates were incubated at 30°C for 5 days. Plates containing fungal plugs without the isolates were used as control. The inhibition zone was measured after the fungal mycelia in the control plates reached the edges of the plates. Growth inhibition was calculated using the formula:

Percentage⁢ of⁢ growth⁢ inhibition=[(C−T)/C]×100

where, *C* is the radial growth of the test pathogen in the control plates (mm), and *T* is the radial growth of the test pathogen in the test plates (mm). The dual-culture assay was conducted twice, on three plates, at each attempt.

### Pot Trial Experiment for Assessment of Colonization, and Growth Stimulating Effect of AL2-14B in Experimentally Inoculated Plants

Micropropagated plantlets of *A. aspera* were raised from the surface sterilized seeds on half strength Murashige and Skoog (MS) medium. The seeds of *A. aspera* were placed in the Petri dishes containing 25 mL sterilized half strength of MS medium and incubated at 60% humidity, 24 ± 2°C and 1000 lux light (16 h light and 8 h dark). After 4 weeks, when the seedlings have cotyledons and roots, they were transferred to freshly prepared MS medium and allowed to grow. After development of extensive root system and with six leaflets, the plantlets were gradually acclimatized to natural environment and finally planted in sterile soil under greenhouse conditions (26 ± 2°C and 70% RH).

Bacteria were grown to the mid-log phase, and harvested by centrifugation (6000 × *g*, 10 min, 24°C). Pellets were washed twice, and suspended in sterilized double distilled water. The suspension was maintained at the OD_600_ of 1.0. To confirm inoculation density and purity, an aliquot of culture was serially diluted in sterile double distilled water and plated on YEMA medium. The plants were inoculated (in triplicate), when a minimum height of 7.5 cm was attained and the stalks were at least 0.5 cm in diameter, which corresponded to 75–80 days after seed germination. A 26-gauge needle attached to a tuberculin syringe containing a bacterial suspension was passed horizontally through the stem just above the first cotyledon leaves of the plant. A 10 μL droplet of suspension was formed at the tip of the needle, which was withdrawn through the plant stem.

AL2-14B was experimented for its colonizing behavior in aerial region of *A. aspera*. It was inoculated, and re-isolated for estimation of population density, from stem and leaves of experimentally inoculated *A. aspera* seedlings. Morphological and physiological characteristics of the re-isolated endophytic bacterium were compared to assure it to be AL2-14B. Isolates were further confirmed to be *P. aeruginosa* AL2-14B based on 16S rRNA sequence similarity. A 10 μL of AL2-14B suspension (OD_600_ = 1.0, which corresponded to 12.9 × 10^5^ CFU/mL), was inoculated into the plant hosts and grown in the greenhouse condition. The bacterial multiplication in the stem and leaves of plants were determined at 3 and 5 days after inoculation (DAI). Lower parts of stems and first leaves were collected from three replicates, weighed, and surface sterilized in 70% ethanol for 15 s, rinsed with double distilled water and macerated with 1 mL of sterile double distilled water. After 20 min, the supernatant were serially diluted and plated on YEMA amended with 25 μg/mL of kanamycin and incubated at 28°C for 48 h. The control plants were inoculated with sterile double distilled water by using the methods used for the experimental plants. Kanamycin was used for screening against any contamination (if any) for AL2-14B, as it was found to be resistant for kanamycin.

Further, the acclimatized plantlets were transferred into bigger pot having diameter of 25 cm^2^ and depth of 20 cm. The pot has the capacity of holding 5 kg of soil:sand (4:1) and kept at randomized block design. The plants were watered every alternative day. The plants were harvested after 150 days and different growth parameters such as shoot length, root length, numbers of leaves, fresh leaf weight, fresh shoot weight, fresh root weight, dry leaf weight, dry shoot weight, dry root weight, and area of the leaf were measured.

### Availability and Uptake of NPK

Availability of nitrogen, phosphorus, potassium (NPK) in soil, for each treatment was analyzed at initial stage, and after 30 days. Effect of AL2-14B on nutrient uptake of *A. aspera* L. was analyzed in the leaves of 30 days old seedlings. Total N was estimated by Kjeldahl digestion method, total P in plant samples was estimated by ammonium molybdate method; whereas K was analyzed by flame photometric method.

### Experimental Design and Statistical Analysis for Pot Trials

The pot trial experiment had two treatments (with and without bacterial inoculation) each with three replicates having five plants in each pot and arranged in a completely randomized design. All data were subjected to one-way analysis of variance (ANOVA) followed by independent *t*-test at *P* < 0.05 using the SPSS 16 software (SPSS Inc). The CFU data were subjected to single factor ANOVA or *t*-test (assuming equal variance) using the SPSS 16 software (SPSS Inc.). The mean ± standard deviation values are presented.

### Scavenging Effect on DPPH Free Radical

The leaves samples were cleaned, dried, and powered with the help of mixer grinder. The powered leaves were extracted with ethanol and water using soxhlet apparatus at 55–85°C for 8–10 h. The free radical scavenging activity of 50% aqueous ethanolic extract of *A. aspera* L. on stable radical 1,1-diphenyl-2-picrylhydrazyl (DPPH) was evaluated by the method of Brand-Williams et al. (1995). Briefly, 2.0 mL of extract at different concentrations (50–250 μg/mL) was mixed with 2.0 mL of DPPH solution in methanol (0.004% w/v). The mixture was allowed to stand at room temperature in dark for 20 min. The mixture was vortexed and then absorbance was recorded at 517 nm. Ascorbic acid was used as a reference standard and control consisted of DPPH solution without extract. The test was performed in triplicate and percentage scavenging of DPPH free radical by extract was calculated using the equation: [(*A*_control_ -*A*_test_) *A*_control_^-1^] × 100. Here, *A*_control_ was the absorbance of control and *A*_test_ was the absorbance in presence of extract or standard. Mean of three determinations was recorded.

### β-Carotene-Linoleic Acid Assay

β-Carotene bleaching assay was done according Wettasinghe and Shahidi (1999). One milliliter of β-carotene solution (0.2 mg/mL in chloroform) was pipetted into a round bottom flask containing 0.02 mL of linoleic acid and 0.2 mL of 100% Tween-20. The mixture was evaporated in a rotary vacuum evaporator for 10 min to remove chloroform. The mixture was immediately diluted with 100 mL of distilled water with vigorous shaking to form an emulsion. Varying concentrations of extract and standard (100–500 μg/mL) was added to 5 mL of the emulsion in different test-tubes and the mixture was kept at 37°C for 1 h. Absorbance of sample and control was measured at time *t* = 0 and *t* = 60 min. Total antioxidant activity was calculated based on the following equation: AA = [1 - (*A*_0_ -*A*_t_) × (*A*_0_^0^ -*A*_t_^0^)^-1^] × 100, where AA is antioxidant activity, *A*_0_ and *A*_0_^0^ are the absorbance values measured at the initial incubation time for samples and control, respectively while *A*_t_ and *A*_t_^0^ are the absorbance values measured in the samples or standards and control at *t* = 60 min. Mean of three determinations was recorded.

### Determination of Reducing Power

The reducing power of leaves extract was determined by the method of Jayanthi and Lalitha (2011). Substances possessing reducing power react with potassium ferricyanide (Fe^3+^) to form potassium ferrocyanide (Fe^2+^) which then reacts with ferric chloride to form ferric ferrous complex that has an absorption maximum at 700 nm. Varying concentrations of plant extract and standard (50–250 μg/mL) were mixed with phosphate buffer (2.5 mL) and potassium ferrocyanide (2.5 mL). The mixture was kept at water bath at 50°C for 20 min. After cooling 2.5 mL of 10% trichloroacetic acid was added and centrifuged at 3000 × *g* for 10 min. The upper layer (2.5 mL) of the resulting solution was mixed with distilled water (2.5 mL) and freshly prepared ferric chloride solution (0.5 mL). The absorbance was measured at 700 nm. Ascorbic acid at various concentration was taken as standard. Increase in absorbance indicated the increase in reducing power of extract as compared to standard. Mean of three determinations was recorded.

## Results

### Isolation and Characterization of Endophytic Isolate AL2-14B

**FIGURE 1:**
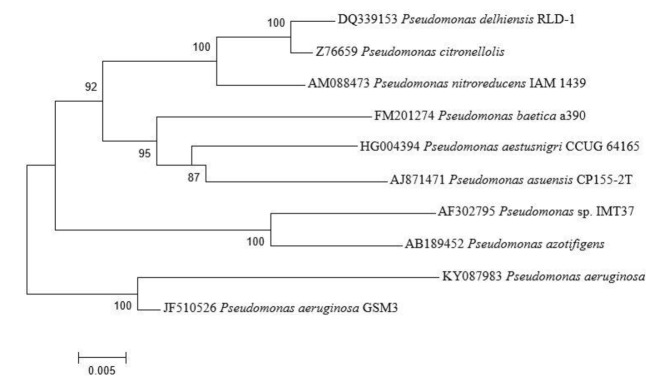
Phylogenetic analysis of 16S rRNA sequences of the bacterial isolate AL2-14B isolated from *A. aspera* L. The analysis was conducted with MEGA6 using neighbor-joining method.

Seventy-three isolates were obtained from different area of Manipur, India, as endophytic bacteria from the leaves and stems of *A. aspera*. One of the isolate, AL2-14B was selected for this study because of its excellent plant growth stimulating attributes. AL2-14B was Gram-negative, catalase and oxidase positive, small rod that forms irregular greenish-brown, circular, and smooth colonies on YEMA. It fermented glucose and lignin but found negative for other carbohydrates such as fructose, mannitol, sucrose, cellulose, and maltose. It was indole negative, methyl red negative, Voges–Proskauer negative and Simmons’ citrate positive. The results of the BLAST analysis of the 1200 bp long 16S rRNA gene sequence indicated that AL2-14B isolate is closely related to *P. aeruginosa*. Based on the phylogenetic tree constructed with the 16S rRNA similarity (%), it was identified as *P. aeruginosa*, and maximum similarity was observed with isolate *P. aeruginosa* JCM 5962T/BAMA01000316 (**Figure 1**). Strain AL2-14B clustered with *Pseudomonas indica* and *P. aeruginosa*. 16S rDNA sequence of strain AL2-14B was submitted to GenBank under accession no. KY0879823.1

The isolate did not show any hemolytic activity on blood agar medium suggesting it to be non-producer of hemolytic cytotoxin. PCR-based screening also confirmed it to be negative for *toxA* and *plcH* genes, which has been suggested as marker for pathogenic strains of *P. aeruginosa* (Sabharwal et al., 2014).

### Siderophore Production Assay

**FIGURE 2:**
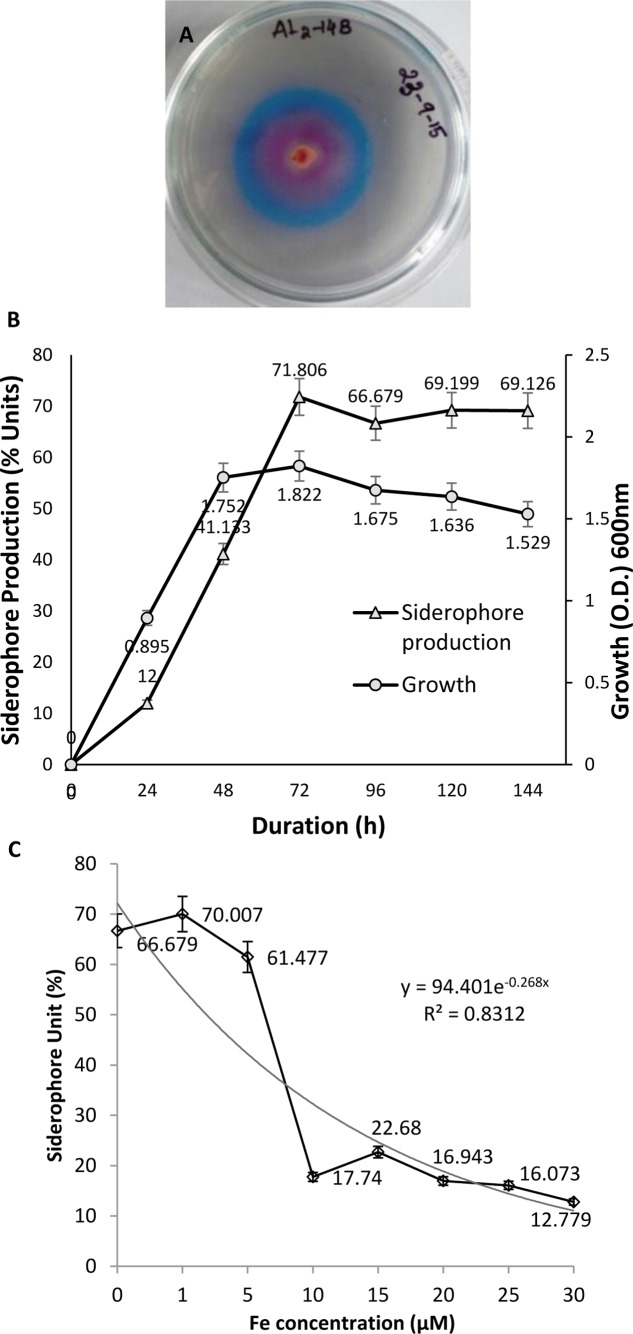
*Pseudomonas aeruginosa* AL2-14B. **(A)** Siderophore release and formation of halozone in CAS agar medium; **(B)** quantitative estimation of siderophore release against growth of bacteria; **(C)** effect of iron on siderophore production.

Formation of orange halo zone in CAS medium inoculated with AL2-14B was observed after 24 h, which indicated the production of siderophore. The zone size increased with time. Zone of siderophore produced at 96 h was found to be 40% higher than that of zone produced at 24 h. Siderophore release was further confirmed by quantitative CAS test where instant decolourization of CAS reagent from blue to orange was observed. A total of 71.806% unit of siderophore was recorded for AL2-14B in succinate broth (**Figure 2A**). In *P. aeruginosa* AL2-14B, the siderophore production was found to start after 24 h of incubation in SM broth and maximum siderophore was released after 72 h of incubation (71.806% units) (**Figure 2B**). It was evident that the siderophore production was high at late log phase, and amount of siderophore release was in accordance with the growth profile of isolate AL2-14B.

#### Effect of Iron on Siderophore Release

Siderophore production was considerably affected by the presence of iron in medium. Initial increase in iron concentration induced siderophore production, but further increase in iron concentration resulted in successive decrease of siderophore production by *P. aeruginosa* AL2-14B. Maximum siderophore release was recorded at 1 μM concentration of iron. Siderophore production decreased to 17.74 SU at 10 μM of iron and remained almost in the same range for higher concentrations of iron tested (**Figure 2C**).

### IAA Production Assay

**FIGURE 3:**
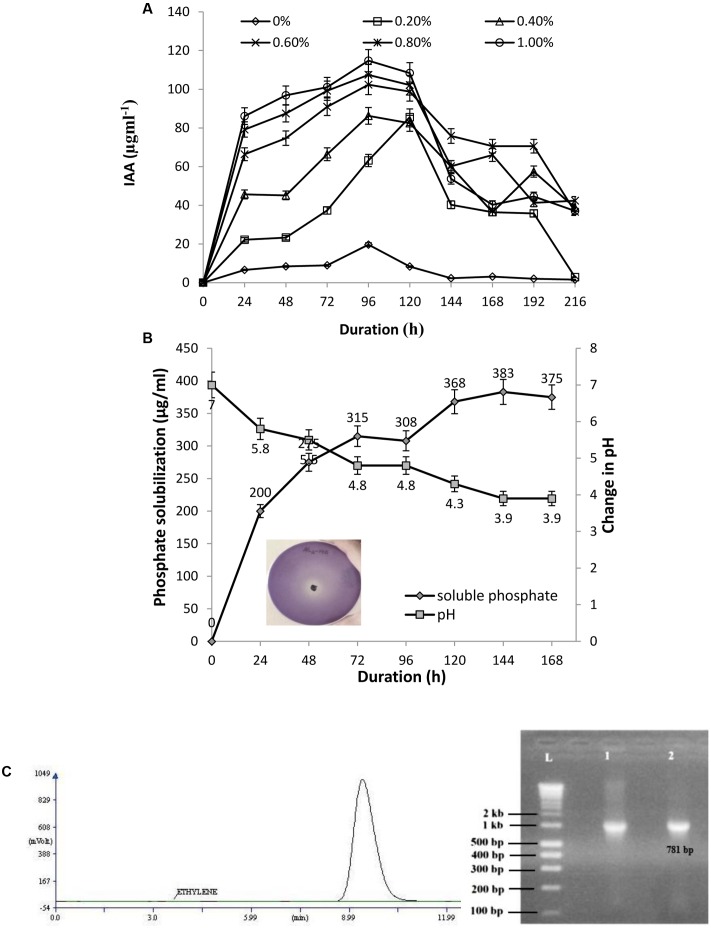
**(A)** Quantitative estimation of IAA produced by AL2-14B at different L-tryptophan concentrations; **(B)** phosphate solubilization by AL2-14B after different time intervals. Soluble free phosphate concentration is given against primary *y*-axis, while variation of pH in the culture medium is given at secondary *y*-axis. Standard deviation showed as bars. Photograph represents the zone of clearance around colony of AL2-14B confirming its role in phosphate solubilization; **(C)** nitrogen fixation: acetylene reduction assay for nitrogenase activity, and *nifH* amplification (L, ladder; lane 1, *Klebsiella pneumoniae* S4C9 as positive control; lane 2, AL2-14B).

The isolate AL2-14B was screened for the ability to produce IAA. Varying levels of IAA production was recorded with different concentration of L-tryptophan (0, 0.2, 0.4, 0.6, 0.8, and 1.0%). The IAA production was in the range of 6.64–114.79 μg/mL. A 1.0% concentration of L-tryptophan was found to be optimum for IAA production by this isolate. IAA production decreased at higher concentrations of tryptophan (**Figure 3A**). The amount of 1AA production was maximum after 96 h of incubation for all the concentrations of tryptophan, except 0.6% (**Figure 3A**).

### Phosphate Solubilization, Nitrogen Fixation, and ACC Deaminase Activity

AL2-14B solubilized tri calcium phosphate in Pikovskaya’s agar, forming a clear halo around the colony (**Figure 3B**). The phosphate solubilization index of the isolate was found to be in the range of 2.33 ± 0.17 to 3.66 ± 0.28, between 24 and 96 h. The pH of the medium decreased with increase in the amount of free phosphate released, showing maximum P solubilization at pH 3.9 after 144 h of incubation (383 μg/mL) (**Figure 3B**). The correlation coefficient (*r*) between free P concentrations against pH after various durations was calculated and found to be (-) 0.9683.

Further, the isolate was found to have nitrogen fixation ability. The growth of AL2-14B in NFCC medium, was observed to be close to surface, but not on surface. The ability to fix nitrogen was further confirmed by quantifying Nitrogenase activity, and it was found to be 1.8617 ± 0.31 nmol ethylene/μg/protein/h as detected using GC-FID technique (**Figure 3C**). Further, N-fixing ability was confirmed by the presence of *nifH* gene, and desired amplicon of 781 bp corresponding to *nifH* gene was obtained in *P. aeruginosa* AL2-14B (**Figure 3C**). The isolate was unable to growth on minimal salt medium amended with ACC, which indicate that AL2-14B did not produce ACC deaminase.

### Determination of Antifungal Activity

**FIGURE 4:**
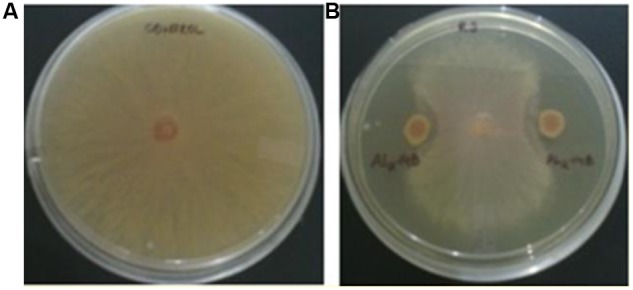
Antagonistic activity of AL2-14B. **(A)** Control, **(B)** dual culture assay for assessment of mycelial growth inhibition of *Rhizoctonia solani* by AL2-14B.

The isolate AL2-14B showed considerable inhibition of mycelial growth, because of release of diffusible compound(s) against *R. solani*. Percentage of growth inhibition was found to be 68.75 ± 2.72% (**Figure 4**). However, there was no zone of inhibition observed with *F. oxysporum* and *P. oryzae*, suggesting limited antifungal activity in AL2-14B.

### Pot Trial Experiment for Assessment of Colonization, and Growth Stimulating Effect of AL2-14B in Experimentally Inoculated Plants

During *in vitro* propagation of *A. aspera* in germ free condition, 29% of seeds germinated in half strength MS medium as compared to full strength MS medium, in which only 22% seeds germinated. Better germination was observed in seeds without husk, as compared to husk coated seeds. The colonization ability of AL2-14B was determined, and it was found that the population of AL2-14B increased from 70 × 10^6^ to 32.3 × 10^10^ CFU/g (fresh weight) between 3rd and 5th DAI (**Table 1**) in stem of seedlings. Further, AL2-14B was not detected in leaves till 3 DAI. However, after 5 DAI, 11.3 × 10^4^ CFU/g of AL2-14B was recovered from leaves of bacteria-treated *A. aspera* plants. The representative control trials yielded no other indigenous bacteria.

**Table 1:** Population of endophytic bacteria from *A. aspera* grown in greenhouse condition.

Bacteria	Initial population of bacteria as inoculated		Final population of bacteria, CFU/g (fresh weight)			
			
			3 DAI		5 DAI	
			
	Stem	Leaf	Stem	Leaf	Stem	Leaf
AL2-14B	12.9 ± 0.25 × 10^5^ (a)	Nil	70 ± 0.51 × 10^6^ (b)	Nil	32.3 ± 0.78 × 10^10^ (c)	11.3 ± 0.32 × 10^4^ (d)


*Values with different letter within a row are significantly different from initial population at *P* < 0.05.*

**FIGURE 5:**
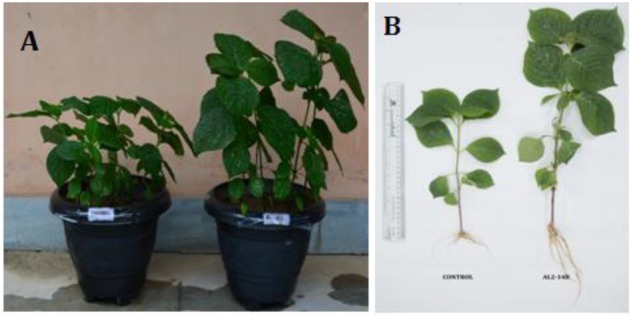
**(A,B)** Effect of *P. aeruginosa* AL2-14B on growth promotion of *A. aspera* L.

Inoculation of endophytic bacteria AL2-14B in host plant resulted increase in all growth parameters of *A. aspera* L. It significantly increased shoot length by 72.83 ± 1.24% (*P* < 0.05), fresh shoot weight by 302 ± 2.74% (*P* < 0.05), dry shoot weight by 486 ± 1.43% (*P* < 0.05), fresh root weight by 385.71 ± 3.69% (*P* < 0.05), dry root weight by 700 ± 3.92% (*P* < 0.05), and area of leaves by 135.28 ± 1.6% (*P* < 0.05) (**Table 2** and **Figure 5**). Further, the NPK concentration, i.e., the availability was estimated in the soil planted with *A. aspera* L. (without inoculation) at the 1st day and after 30th day of the treatments. It showed that the NPK concentration in soil at the 1st day was 42 ± 0.63, 35.42 ± 2.11, and 80.5 ± 2.38 mg/kg, respectively, which was found to decreased in inoculated with AL2-14B. The NPK content in soil in which AL2-14B was inoculated with plants, decreased up to 36 ± 0.13, 33 ± 0.86, and 39.2 ± 0.5.11 mg/kg, respectively. Again, the uptake of NPK was analyzed by estimating their concentration in the leaves. In the case of control plant (without inoculation), the NPK content in leaves was found to be 29,400 ± 121, 2300.85 ± 24.2, and 48,550 ± 234 mg/kg, respectively. The NPK content in leaves with AL2-14B was significantly higher, recorded as 30,520 ± 320 mg/kg (*P* < 0.05), 2589.64 ± 64 mg/kg (*P* < 0.05), and 57850 ± 199 mg/kg (*P* < 0.001), respectively. The plants were grown till 150 days for estimation of growth parameters.

**Table 2:** Effect of *P. aeruginosa* AL2-14B on the growth characteristics of the *A. aspera* L.

Parameters	Control	Inoculated with AL2-14B
Shoot length (cm)	20.54 ± 2.5 (a)	35.50 ± 0.93 (b)
Root length (cm)	10.20 ± 2.28 (a)	22.86 ± 1.63 (a)
Number of leaves	9.60 ± 0.89 (a)	20.2 ± 0.83 (a)
Fresh leaves weight (g)	0.26 ± 0.12 (a)	0.50 ± 0.12 (a)
Dry leaves weight (g)	0.05 ± 0.023 (a)	0.12 ± 0.03 (a)
Fresh shoot weight (g)	0.84 ± 0.14 (a)	3.38 ± 0.64 (b)
Dry shoot weight (g)	0.15 ± 0.02 (a)	0.88 ± 0.43 (b)
Fresh root weight (g)	0.21 ± 0.35 (a)	1.02 ± 0.53 (b)
Dry root weight (g)	0.04 ± 0.005 (a)	0.32 ± 0.17 (b)
Area of leaves	24.77 ± 2.11 (a)	58.28 ± 5.95 (b)


*Each value is the mean of five plants. Values with the same letter within a row are not significant at *P* < 0.05.*

### Determination of Antioxidant Activity—Scavenging Effect on DPPH Free Radical, β-Carotene-Linoleic Acid Assay, and Determination of Reducing Power

**FIGURE 6:**
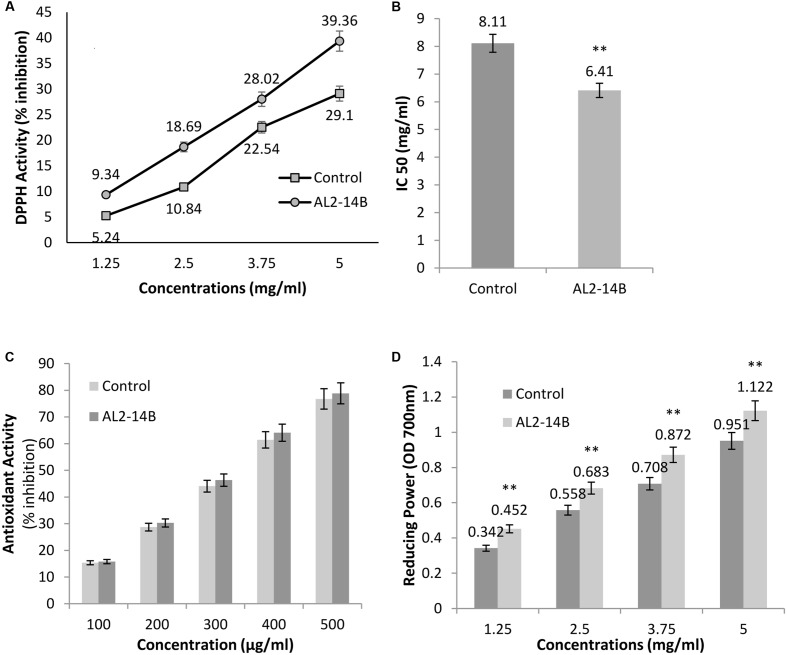
**(A)** The free radical scavenging activity with DPPH, against different concentrations of *A. aspera* L. extract; **(B)** concentration of samples that scavenge 50% free radicals of DPPH, expressed as IC_50_ (mg/mL); **(C)** antioxidant activity of samples using β-carotene bleaching assay; and **(D)** reducing power of samples at 700 nm. All results are expressed as mean ± SD of three determinations. ^∗∗^Significant at *P* < 0.05.

*Achyranthes aspera* L. plant treated with the isolate AL2-14B showed higher DPPH radical scavenging activity compared to control plant (**Figure 6A**). The free radical scavenging activity of the extract was concentration dependent. The values of DPPH activity of treated plant ranged from 9.34 ± 2.12 to 39.36 ± 3.26 while the values of DPPH activity of control plants were in the range of 5.24 ± 1.28 to 29.1 ± 2.52. IC_50_ was observed at a concentration of 6.41 ± 0.11 mg/mL for DPPH free radical scavenging activity of AL2-14B with the control plant having IC_50_ of 8.11 ± 0.24 mg/mL (**Figure 6B**).

β-Carotene-linoleic acid content of extract was found to be more in AL2-14B inoculated plants as compared to control. However, the difference in the activity was not significant (*P* < 0.05). In fact, the activity of the plant treated with AL2-14B ranged from 15.77 to 78.85 while that of un-inoculated control plant were in the range of 15.35–76.76 (**Figure 6C**).

The reducing power of the plant extract increased with respective increase in concentration. The reducing power of *A. aspera* L. leaves inoculated with AL2-14B was found to be higher than that of control plant. The values ranged from 0.452 to 1.122 in inoculated plant but in control plant the values were found in the range from 0.342 to 0.951. For each concentration of extract, the reducing power was found to be significantly higher (*P* < 0.05). The results for reducing power activity of *A. aspera* leaves extract are given in **Figure 6D**.

## Discussion

Endophytes had been defined in “microbes that settle living, in interior tissues of plants without causing any instant, exert negative effects” (Bacon and White, 2000), but recently, this definition has been elaborated where endophytes have been suggested “to have both positive and negative effect on the plant, as well as they can be neutral to the plant” (Brader et al., 2017). A siderophore producing diazotrophic endophytic *P. aeruginosa* AL2-14B was isolated from the leaves of *A. aspera* L. Though, a phosphate solubilizing *P. aeruginosa* EPR13 has been reported from the root of *A. aspera* L. (Misra et al., 2012), there has been no earlier reports on exclusive dominance of diazotrophic *P. aeruginosa* endophyte in aerial tissues that affects growth, and antioxidant activities in *A. aspera*, as described in this report.

Siderophores produced by bacteria contribute in enhancing the growth and yield of agricultural plants. *P. aeruginosa* AL2-14B produced significant amount of siderophore. In fact, 71.806% unit of siderophore was recorded in succinate broth after 72 h of incubation. The pattern of siderophore release was correlated with growth, and results were in accordance with similar study on siderophore production reported in *Azospirillum* (Saxena et al., 1986), where maximum siderophore production was recorded after 20 h of growth. Pyoverdine type siderophore in *P. aeruginosa* PAO1 was also found to be released in maximum amount after 40 h of growth (Barbhaiya and Rao, 1985). Similarly, maximum siderophore production was recorded at the time of commencement of late log phase, which occurred parallel with growth, as observed in previous work (Barbhaiya and Rao, 1985). In bacteria, it has been reported that starvation of iron in medium stimulates siderophore production (Pandey et al., 2005). Therefore, a very low amount of iron was found to induce siderophore production. Further, there was steady decrease in siderophore release, with increase in iron concentrations, which indicate that the siderophore production in AL2-14B is under strict control of iron concentration. The most suitable iron concentration for siderophore production unit was 1 μM. Sayyed et al. (2005) also found maximum siderophore production at 1 μM of iron by *P. fluorescens* NCIM 5096 and *P. putida* NCIM2847. In this study, both the Arnow’s and FeCl_3_ tests were positive, revealing the catecholate and hydroxamate type of siderophores. In earlier reports, endophytic *P. aeruginosa* PM389, isolated from the healthy pearl millet plant produced catecholate type of siderophore (63.5% units) with 0.711 siderophore index (Gupta et al., 2013). Pandey et al. (2005) reported that *P. aeruginosa* GRC1, isolated from mustard plant, produced 18.76 μg/mL of hydroxamate type of siderophore at the iron concentration of 0.2 μM.

The isolate *P. aeruginosa* AL2-14B was found to release appreciable amount of IAA (114.79 μg/mL), which was induced by L-tryptophan, suggesting that this isolate has tryptophan dependant IAA release mechanism. Maximum IAA was released after 96 h of incubation, which was in agreement with earlier reports (Sachdev et al., 2009; Khamna et al., 2010). Kumar et al. (2016) reported that *P. putida* ECL5 produced 23 μg/mL of IAA on supplementation of 400 μg/mL of L-tryptophan after 48 h of incubation. Fouzia et al. (2015) also reported that *P. fluorescens* CHAO, *P. fluorescens* RB13, and *P. aeruginosa* EH4 produced 88.37, 50.95, and 36.88 μg/mL of IAA after 48 h of incubation, respectively, with supplement of 5 g/L of L-tryptophan.

It is known that bacteria can solubilize inorganic phosphate by releasing organic acids. The carboxylic group of organic acids chelate the cations (mainly Ca) bound to phosphates, and thus converting them into the soluble forms (Kpomblekou and Tabatabai, 1994). The isolate *P. aeruginosa* AL2-14B formed halo zone when inoculated on the Pikovskaya’s solid medium containing tri-calcium phosphate. The pH of the medium decreased with the increase in the amount of free phosphate released, showing maximum P solubilization at pH 3.9 after 144 h of incubation (383 μg/mL). Similar results were reported earlier by Kaur and Reddy (2013) with two rhizobacterial isolates of *Stevia rebaudiana*, namely *Pantoea cypripedii* PSB-3 and *Pseudomonas plecoglossicida* PSB-5, which were found to solubilize 253 mg/mL and 271 mg/mL of inorganic phosphate, respectively after 5 days of incubation.

Endophytic *P. aeruginosa* AL2-14B was unable to grow on the surface of NFCC medium, but turbidity was observed in deeper regions of culture tubes. This indicates that AL2-14B may fix nitrogen in low oxygen or anaerobic environment only. Earlier, Mirza and Rodrigues (2012) have isolated free living diazotrophic pseudomonads from soil by maintaining hypoxic conditions, and further confirmed it by nitrogenase assay. In acetylene reduction assay, nitrogenase activity was recorded to be 1.8617 nmol ethylene/μg/protein/h, as estimated using GC-FID technique. Nitrogenase assay is considered as confirmatory test for estimation of nitrogen fixation which is an important attribute for endophytic PGPR. Diazotrophic nature of AL2-14B was further confirmed by the presence of *nifH* gene element. The presence of *nifH* in endophytic *P. aeruginosa* has been detected by PCR in previous studies (Gupta et al., 2013). N-fixing endophytic bacteria are considered better than their rhizospheric and rhizoplanic counterparts as they provide fixed nitrogen directly to their host (Cocking, 2003). Moreover, endophytic bacteria are less vulnerable to competition with other soil microbes for scarce resources and remain protected to various abiotic and biotic stresses (Reinhold-Hurek and Hurek, 1998). Earlier, Taule et al. (2012) and Reinhardt et al. (2008) had reported N-fixing endophytic *Pseudomonas* spp. from sugarcane and maize plants, respectively.

*Rhizoctonia solani* is a well-known fungal pathogen that causes root rot of several plants. Endophytic bacteria AL2-14B was found to have the ability to inhibit the mycelia growth of pathogenic fungus *R. solani*. Pseudomonads are known for their biocontrol potential against phytopathogens. Earlier, it has been reported that the metabolites produced by *P. aeruginosa* MML2212 inhibited the mycelia growth of *R. solani* (Shanmugaiah et al., 2010). Also, *Pseudomonas* sp. was used as biocontrol agents for controlling broccoli root rot disease caused by *R. solani* pathogen (El-Mohamedy et al., 2011). This property further improves the utility of *P. aeruginosa* AL2-14B in plant growth, which may be useful in *in situ* biocontrol of *R. solani*, and need further study. Recently, Egamberdieva et al. (2017) isolated endophytic bacteria from two medicinal plants, *Hypericum perforatum* and *Ziziphora capitata* and concluded that antimicrobial activity of medicinal plants is improved by the presence of antagonistic endophytes. The colonization of the bacteria in the host tissues also varied with the plant parts. *P. aeruginosa* AL2-14B was inoculated in stem tissues, and gradual increase its population was observed in stem. Till 1st 3 days, leaves were not populated by the endophyte, however, after 5 DAI, the introduced bacteria were found both in stem (21 × 10^6^ CFU/g, fresh weight) and leaves (11.3 × 10^4^ CFU/g, fresh weight) in good numbers. It seems that the bacteria were translocated from stem to leaves through transpiration. This hypothesis is supported by Compant et al. (2005) where he reported that, *Burkholderia* sp., an endophyte of *Vitis vinifera*, spread to aerial parts of host plant through the transpiration stream. The upward passive migration of endophytic bacteria possibly through transpiration stream in xylem vessels of stems has been suggested by other workers too (Thorne et al., 2006). However, there are only few reports on experimental greenhouse study of colonization of host plants by bacterial endophytes. For example, *P. aureofaciens* was inoculated and was recovered after 29 days from tall fescue leaves and pea and bean stems, with population of 2.3 log10 CFU/g (fresh weight) (Lamb et al., 1996). Fisher et al. (1992) reported colonization levels of 2.3–6.5 log CFU/g (fresh weight) of bacteria recovered from field-grown sweet corn. Similarly, Guo et al. (2002) inoculated 4.55 log CFU/mL of salmonellae in the roots of tomato plants grown in hydroponic medium, and found that around 3 log CFU/g of salmonellae were present in hypocotyls, cotyledons, and stems after 24 h. Though, there is no earlier report on colonization of diazotrophic pseudomonads in aerial tissues of *A. aspera*, the results suggest that its endophytic existence has significant role in overall physiology of *A. aspera*.

There are few reports, where *Pseudomonas* sp. has been studied for growth enhancement of *Achyranthes*. However, these isolates were reported from rhizospheric soil of *A. aspera* (Mohinder et al., 2011; Misra et al., 2012). Here we report an endophytic *P. aeruginosa*, which was isolated from the aerial part of the plant, and promote the growth of the host plant as confirmed by pot trial experiments. The results of pot trials suggest that AL2-14B is an excellent growth promoter of *A. aspera*, as significant increase in growth parameters of plants was recorded. AL2-14B significantly (*P* < 0.05) increased shoot length by 72.83%, fresh shoot weight by 302%, dry shoot weight by 486%, fresh root weight by 385.71%, dry root weight by 700%, and area of leaves by 135.28%. Earlier, *Pseudomonas* spp. have been reported as endophytic plant growth promoter in few other plants (Dalal and Kulkarni, 2013; Jasim et al., 2013), but not in *A. aspera*. The treatment of plants with endophytic *P. aeruginosa* AL2-14B increased NPK contents in plant by 3.8, 12.59, and 19.15%, respectively, which was further supported by reduction in NPK content of soil in which these plants were grown. Therefore, endophytic colonization of AL2-14B also improved the nutrient uptake. The higher values of N in plants inoculated with AL2-12B may be attributed to its diazotrophic functions (Santoyo et al., 2016). In this study, the soil was not supplemented with any kind of chemical fertilizers or pesticide. Therefore, the growth stimulation of host plants, as compared to control, may be credited to the endophytic colonization of *P. aeruginosa* AL2-14B, and its various plant growth stimulating attributes including IAA synthesis, nitrogen fixation, and siderophore production. As a matter of fact, mechanism of plant growth stimulation by interactions of endophyte has been attributed to collective effects of different physiological properties of bacteria, including the production of phytohormones, siderophores, and antifungal compounds (Santoyo et al., 2016), which is also valid for *P. aeruginosa* AL2-14B.

In spite of concerns related to opportunistic infections caused by *P. aeruginosa*, it is an established plant growth promoting bacteria. *P. aeruginosa* is known to stimulate growth of host plant indirectly by biocontrol of phytopathogens (Shanmugaiah et al., 2010), induced systemic resistance (Audenaert et al., 2002); or directly by producing siderophore, IAA (Pandey et al., 2005). AL2-14B does not have cytotoxin producing ability and also, it lack two crucial virulent genes. Earlier, Radhapriya et al. (2015) excluded the concern of pathogenicity in *P. aeruginosa* strain RRALC3 isolated from rhizospheric soil by screening for virulence genes *ecfX*, *lasB*, and *ybtQ*.

The antioxidant potentials of *A. aspera* L. substantiate its role as an anticancer agent. Endophytic bacteria help in the mediation of reactive oxygen species and antioxidant activity in plants. Effect of endophytic bacteria AL2-14B was determined on antioxidant activity of *A. aspera* by undertaking several experimental parameters including DPPH scavenging activity, β-Carotene-linoleic acid assay, and reducing power of plant extract. It was observed that inoculation of endophytic isolate *P. aeruginosa*, the antioxidant activity of host plant was increased. It has been suggested earlier, that there is a significant impact of endophyte colonization on the antioxidant activity in plants, as antioxidant activities are higher for colonized in comparison to non-colonized host plants (Hamilton et al., 2012). In fact, antioxidants serve to transmit stress signals through the interaction of oxidant and antioxidant (Foyer and Noctor, 2005), which has been suggested to facilitate the chemical communication between the plant and its endophytic symbiont. This process allows the host plant to differentiate a pathogen from a mutualist, and respond accordingly (Hamilton et al., 2012). There are several reports where endophyte colonization of host tissues has been found to alter the production of antioxidants in plants. High antioxidant activities were recorded when *Phyllosticta* sp. was exposed to reactive oxygen species (Srinivasan et al., 2010). It was suggested that the interaction between endophytic bacteria and host plant affects the host’s hypersensitive and systemic acquired resistance responses, which may be mediated by the production of reactive oxygen species and antioxidants (Tanaka et al., 2006). DPPH assay is vital to assess free-radical scavenging ability of antioxidants. In fact, hydrogen-donating ability is an important attribute of the primary antioxidants, which donate hydrogen to free radicals, resulting in formation of non-toxic species (Lugasi et al., 1998; Sánchez-Moreno et al., 1999). In this study, DPPH activity of the treated plant was found to be higher than the control plant. The free radical scavenging activity of the extract increased with increased in concentration. IC_50_ of the inoculated plant was observed at 20.96% lower than that of control plant. β-Carotene-linoleic acid assay of extract obtained from inoculated plant was found to be slightly higher than the control plant. This assay is based on reaction of β-carotene with radicals formed by linoleic acid oxidation, resulting in loss of the yellow color. Therefore, low rate of beta carotene bleaching is indicative of presence of antioxidants.

Reducing power serve as a good indicator of antioxidant potential of the plant. Compounds having reducing power are actually electron donors, which reduce the oxidized intermediates of lipid peroxidation. The reducing power of *A. aspera* L leaves inoculated with AL2-14B was found to be significantly higher than that of control plant. A total of 23 and 18% increase in the reducing power of the extract were recorded at 3.75 and 5 mg/mL of extract concentration, respectively. In a different study, Prasad et al. (2013) reported that the medicinal plant *Bacopa monnieri* treated with root endophytes *Piriformo sporaindica* DSM 11827 produced higher amounts of bacosides antioxidants than untreated control. The endophyte *P. indica* significantly increased the amount of ascorbic acid and elevated the activities of antioxidant enzymes in barley root under stress conditions (Baltruschat et al., 2008). Therefore, endophytic existence of *P. aeruginosa* AL2-14B was detrimental in affecting the growth of *A. aspera* L, that may be attributed to its growth stimulating attributes like N-fixation, IAA synthesis, siderophore production, and strong antagonistic activity against pathogenic fungus. *P. aeruginosa* AL2-14B also induced antioxidant activities and therefore enhanced the medicinal value of host plant, which present the prospects of *P. aeruginosa* AL2-14B to be utilized as a biofertilizer in the future. Recently, Song et al. (2017) has reported that *Bacillus altitudinis* strain LB 5-3, an endophyte isolated from *Panax ginseng* increases biomass of host plant and improves the medicinal value of plant by eliciting ginsenoside accumulation. Similarly, inoculation of diazotrophic endophytic *P. aeruginosa* was shown to increase in plant growth in *Pennisetum glaucum* (L.) R. Br. (Gupta et al., 2013), however, here we report it for *A. aspera*. Role of endophytic bacteria in enhancement of the antioxidant activity of *A. aspera* has not been reported earlier.

## Conclusion

*Pseudomonas aeruginosa* AL2-14B, an endophytic isolate from the aerial part of the *A. aspera* L. plant produced good quantity of siderophore, IAA, and solubilized inorganic phosphate. It enhances the growth and antioxidant properties of the host plant. Therefore, isolate AL2-14B have potential to be utilized as a biofertilizer for improvement of plant growth. Because the bioformulations based on endophytic organisms are considered as environment friendly alternative to agrochemicals, AL2-14B can be exploited as a growth promoting agent. Further, improved antioxidant activity in *A. aspera* L. is added advantage for the value addition of this medicinal plant.

## Author Contributions

KD have performed the growth and symbioses related experiments, wrote manuscript. GP and AR have contributed for antioxidant activities, GS provided important research inputs, PP has designed the work, formulated strategies, and wrote manuscript.

## Conflict of Interest Statement

The authors declare that the research was conducted in the absence of any commercial or financial relationships that could be construed as a potential conflict of interest.
